# Adverse Childhood Experiences and Their Relationship with Poor Sexual Health Outcomes: Results from Four Cross-Sectional Surveys

**DOI:** 10.3390/ijerph19148869

**Published:** 2022-07-21

**Authors:** Sara K. Wood, Kat Ford, Hannah C. E. Madden, Catherine A. Sharp, Karen E. Hughes, Mark A. Bellis

**Affiliations:** 1 Policy and International Health, World Health Organization Collaborating Centre on Investment for Health and Well-Being, Public Health Wales, Wrexham LL13 7YP, UK; karen.hughes18@wales.nhs.uk (K.E.H.); m.a.bellis@bangor.ac.uk (M.A.B.); 2 Public Health Collaborating Unit, School of Medical and Health Sciences, College of Human Sciences, Bangor University, Wrexham LL13 7YP, UK; k.ford@bangor.ac.uk; 3 School of Social Sciences, Liverpool Hope University, Hope Park, Liverpool L6 1HP, UK; maddenh@hope.ac.uk; 4 Applied Sports, Technology, Exercise and Medicine Research Centre, Faculty of Science and Engineering, Swansea University, Swansea SA1 8EN, UK; catherine.sharp@swansea.ac.uk

**Keywords:** adverse childhood experiences, sexual health, teenage pregnancy, sexually transmitted infections, risky sexual behaviour, trauma-informed services, prevention

## Abstract

Improving understanding of risk factors for risky sexual behaviour is fundamental to achieve better population sexual health. Exposure to adverse childhood experiences (ACEs) can increase the risk of poor sexual health outcomes, but most research is US-based. This study explored associations between ACEs and poor sexual health outcomes in the UK. Data from four cross-sectional ACE surveys with adult general populations in different regions of the UK from 2013–2015 (*n* = 12,788) were analysed. Data included participants’ demographics, ACE exposure, and four sexual health outcomes: having early sex (<16 years), having an accidental teenage pregnancy, becoming a teenage parent, or having a lifetime diagnosis of a sexually transmitted infection. ACE count was a consistent and significant predictor of all four sexual health outcomes for both males and females, with odds of these outcomes between three and seven times higher for those with 4+ ACEs compared to those with no ACEs. Increased risks of some, but not all, sexual health outcomes were also found with higher residential deprivation, younger age, being of white ethnicity, and being born to a teenage mother. Findings highlight the need for effective interventions to prevent and ameliorate the lifelong effects of ACEs. Trauma-informed relationships and sex education, sexual health services, and antenatal/postnatal services, particularly for teenagers and young parents, could provide opportunities to prevent ACEs and support those affected. Ensuring that those living in deprived areas have access to services and that barriers to uptake are addressed is also key.

## 1. Introduction

Improving population sexual health is an important worldwide public health goal [1]. Globally, more than one million sexually transmitted infections (STIs) are thought to be acquired each day [2], whilst an estimated 15% of women give birth before the age of 18 [3]. Whilst rates of teenage births are much lower in high-income compared to low-income countries (11 and 93 per 1000 population, respectively, in 2019; [4]), teenage pregnancy and related sexual health issues such as unplanned pregnancies, abortions, and transmission of STIs remain problematic [2,5].

STIs can present a number of important long-term health implications, including infertility [6], cervical and prostate cancer [7,8], and pelvic inflammatory disease [9]. They can further impact the health of unborn children through higher risk of low birth weight, preterm birth, or stillbirth [10,11,12]. Teenage pregnancy has also been associated with pregnancy complications [13] and poorer socio-economic outcomes for both mother and child [14], whilst unplanned pregnancy can increase the risk of later child maltreatment [15]. Poor sexual health places a considerable financial burden on health and other public sector services. For instance, in the US in 2018, STIs (including human immunodeficiency virus [HIV]) were estimated to cost the healthcare system almost USD 16 billion [16].

Understanding the risk factors and pathways that lead to risky sexual behaviour and poorer sexual health outcomes is fundamental to improving population sexual health. One potential risk factor for poor sexual health outcomes is an individual’s exposure to adverse childhood experiences (ACEs). ACEs are potentially traumatic events occurring in childhood. These vary in severity, but include being a victim of abuse or neglect, or being exposed to adversity within living environments such as domestic violence, or parental substance abuse, mental illness, or incarceration. Since the 1990s, a growing body of international evidence has identified the impacts that ACEs can have on an individual’s neurological and biological functioning [17,18], as well as on a wide variety of poorer health outcomes across the life course. These include greater susceptibility to risky behaviours such as smoking and heavy alcohol use; mental ill health and suicidal behaviours; chronic physical conditions such as cancer, heart disease, and respiratory disease; and premature mortality [19,20,21]. Meta-analyses of international literature suggest that adults exposed to four or more (4+) ACEs (compared to those not exposed) are over three times more likely to report early sexual initiation (before age 16), four times more likely to report a teenage pregnancy (younger than 18/19 years), over three times more likely to report having multiple (five or more) sexual partners, and almost six times more likely to report a lifetime diagnosis of an STI [21]. Other related poor outcomes include early puberty [22], unprotected sex [23,24], and repeat abortions [25]. The associations between ACEs and poor sexual health outcomes appear to be stronger for women compared to men, and for sexual minority groups compared to heterosexuals [26].

Whilst our understanding of ACEs and poor sexual health outcomes is improving, much evidence on this relationship is derived from US-based studies; far less research has been conducted in European countries or elsewhere, where there may be cultural or service provision differences. To address this gap, this study used data from four large surveys of ACEs and health with general adult populations in the UK to explore associations between ACE exposure and four sexual health outcomes: having early sex (before age of 16), having an accidental pregnancy before the age of 18, becoming a teenage parent (aged 18 or less), and having a lifetime diagnosis of an STI.

## 2. Materials and Methods

Data were derived from four cross-sectional ACE surveys conducted between 2013 and 2015 across different regions of the United Kingdom (England [national], Wales [national], South of England, North West England; see Table S1). All four surveys used a random stratified sampling methodology with a lower super output area (LSOA, geographic areas with a mean population of 1600) as the sampling unit, stratified first by region (for national datasets only), and then within region by residential deprivation quintile (using rankings in the English and Welsh indexes of multiple deprivation [IMD]; see Table S1). The IMD is a composite measure which standardises the comparison of deprivation between localities, although there are some differences between the English and Welsh IMD methodologies. For the English surveys, the national postcode address file (PAF) was used to randomly select households of residence in sampled areas who then received letters outlining the study, when researchers might visit, and contact details to opt out. In the Welsh survey, researchers randomly selected addresses within each sampled area, where letters were provided at the doorstep. All four surveys used professional market research companies to undertake data collection, who were provided with training on ACEs prior to data collection.

For all surveys, data were collected face-to-face at the respondents’ places of residence using a combination of computer-assisted personal interviewing (CAPI) and computer-assisted self-interviewing (CASI) for more sensitive questions. Where requested, paper-based questionnaires were provided and surveys were available to be completed in multiple languages depending on the study, including Balochi, Bengali, Hindi, French, Gujarati, Marathi, Pashto, Polish, Punjabi, Saraiki, Sindhi, Spanish, Urdu, and Welsh.

All potential participants were provided with an information sheet outlining the study purpose and its anonymous and voluntary nature. Participants were given the opportunity to opt out or for interviewers to call back at a time more suitable to them. It was made clear to all potential participants that they were free to withdraw at any time and that doing so, or declining participation, would not influence any future treatment or service provision. Study eligibility criteria were: aged 18–69 (or 18–70 in North West England); resident in the selected household; and cognitively able to complete the survey. Only one person per household was invited to take part, with the next birthday rule applied where there were multiple household residents. Across all four surveys, 13,161 surveys were completed. Weighted average compliance across studies was 55.7% (Table S1 reports response rates for individual samples). Individuals who did not complete all questions on demographics (for consistency across samples, the North West England sample was limited to those aged 18–69), individuals who could not be allocated an ACE count, and individuals who did not provide data for at least one sexual health outcome measured were excluded from the analysis. Thus, a final sample of 12,788 participants was used for analyses.

All measures were self-reported. Using 11 questions from the Centers for Disease Control and Prevention (CDC) short ACE tool (see Table S2), participants were asked about their exposure to nine ACE types before the age of 18 years (physical, emotional, and sexual abuse, as well as exposure to household member drug use, alcohol abuse, mental illness, domestic violence, incarceration, and parental separation). Consistent with international literature, an individual’s ACE count was calculated and categorised into four groups for analysis (0, 1, 2–3, or 4+ ACEs). Since daughters of teenage mothers have an increased risk of becoming a teenage parent themselves [27], respondents were asked how old their mother was when they were born. Those whose mothers were aged 18 or less at the time of their birth were categorised as being born to a teenage mother (dichotomised to Yes/No). Surveys measured four sexual health outcomes (See Table S2 for questions and qualifying responses): (1) early sex: if the respondent had sexual intercourse for the first time under the age of 16 (Yes/No); (2) accidental teenage pregnancy: if the respondent had (or caused) an accidental teenage pregnancy before the age of 18 (Yes/No); (3) teenage parent: if the respondent’s first child was born when the respondent was aged 18 or less (Yes/No); and (4) lifetime STI diagnosis: if the respondent had ever been told by a doctor or nurse that they had an STI (Yes/No). Respondent age (18–29, 30–39, 40–49, 50–59, 60–69), gender (male/female), and ethnicity (using UK census categories and categorised for analysis as white/Asian/other due to small numbers in some ethnic groups) were also recorded. Although ethnicity analysis was limited in categories, the proportion of participants who were white (84%) was generally representative of the UK population [28].

Statistical analyses used SPSS v24.0 (IBM, Armonk, NY, USA). Initial analysis used the chi-squared test to explore univariate relationships between the four sexual health outcomes and age, gender, ethnicity, deprivation, ACE count, being born to a teenage mother, and study location. For each sexual health outcome, binary logistic regression (backward stepwise regression) was then used to examine independent relationships with ACE count controlling for age, gender, ethnicity, deprivation, being born to a teenage mother, and study location.

## 3. Results

Just over half (54.9%) of participants were female, and most (83.6%) described themselves as white (Table 1). Less than one in ten (7.2%) reported being born to a teenage mother. A sixth of participants (16.5%) had engaged in early sex, 7.0% reported an accidental teenage pregnancy, 5.6% reported becoming a teenage parent, and 1.8% reported having a lifetime STI diagnosis. Univariate analyses highlighted significant differences in sexual health outcomes across genders (Table 1), with males being more likely to have experienced early sex (19.0% vs. 14.5%, *p* < 0.001), and females being more likely to have experienced accidental teenage pregnancy (9.0% vs. 4.7%, *p* < 0.001) and becoming a teenage parent (8.5% vs. 2.0%, *p* < 0.001). Significant differences were also found across other demographics and the study location (Table 1). A significantly higher proportion of those born to a teenage mother (than those who were not) reported having early sex (26.4% vs. 16.2%, *p* < 0.001), having an accidental teenage pregnancy (13.7% vs. 7.0%, *p* < 0.001), and being a teenage parent (15.9% vs. 4.8%, *p* < 0.001; Table 1).

All sexual health outcomes were significantly associated with ACE count. Prevalence of outcomes increased with ACE exposure and was between three and seven times higher for those reporting 4+ ACEs compared to those reporting no ACEs (Table 1). A significantly higher proportion of those with 4+ ACEs (compared to those with 0 ACEs) reported: early sex (38.6% vs. 10.3%, *p* < 0.001), accidental pregnancy (21.1% vs. 3.9%, *p* < 0.001), becoming a teenage parent (13.4% vs. 3.9%, *p* < 0.001), and a lifetime STI diagnosis (6.7% vs. 0.8%, *p* < 0.001) (Table 1).

Due to significant differences in outcomes between males and females, regression analyses were run separately for each gender. ACE count remained a consistent and significant predictor of all sexual health outcomes for both males (Table 2) and females (Table 3). Across genders, odds of the four outcomes ranged between 3.2 and 7.6 times higher for those with 4+ ACEs compared to those with no ACEs. Thus, compared to those with no ACEs, those with 4+ ACEs were: 3.2 (male, *p* < 0.001) and 4.3 (female, *p* < 0.001) times more likely to report early sex; 6.2 (male, *p* < 0.001) and 4.3 (female, *p* < 0.001) times more likely to report accidental teenage pregnancy; 3.2 (male, *p* < 0.001; female, *p* < 0.001) times more likely to report having been a teenage parent; and 7.6 (male, *p* < 0.001) and 7.1 (female, *p* < 0.001) times more likely to report a lifetime STI diagnosis (Table 2 and Table 3).

Age group remained significant for certain outcomes (male: early sex only; female: early sex, accidental teenage pregnancy, a lifetime STI diagnosis), with the odds for these outcomes being 1.7–6.3 times higher among the youngest age group (18–29 years) compared to the oldest (60–69 years; Table 2 and Table 3). Ethnicity also remained significant for a number of outcomes (male: early sex, accidental teenage pregnancy; female: early sex, accidental teenage pregnancy, teenage parent). For these significant outcomes, participants of white ethnicity were between 1.6 (female: teenage parent) and 17.5 (female: early sex) times more likely to report their experience compared to participants of Asian ethnicity.

Relationships with deprivation were less consistent. For males, those living in the most deprived communities were twice as likely to have had early sex (*p* < 0.001) and 4.5 times as likely to have been a teenage parent (*p* < 0.001) compared to those in the least deprived (Table 2). For females, residents in the most deprived communities were 1.8 times more likely to report accidental teenage pregnancy (*p* < 0.001) and 6.4 times more likely to have been a teenage parent (*p* < 0.001) than those living in the least deprived communities (Table 3). Finally, compared to those that were not, those born to a teenage mother were 1.6 (male, *p* = 0.002) and 1.7 (female, *p* < 0.001) times more likely to report early sex and 2.8 (male, *p* < 0.001) and 2.9 (female, *p* < 0.001) times more likely to report having been a teenage parent. For females only, those born to a teenage mother were also 1.9 (*p* < 0.001) times more likely to have had an accidental teenage pregnancy. However being born to a teenage mother was not associated with having a lifetime STI diagnosis for either gender (Table 2 and Table 3).

## 4. Discussion

Exposure to ACEs is associated with a greater likelihood of early sex, accidental teenage pregnancy, becoming a teenage parent, and having a lifetime STI diagnosis. We found that risks for these sexual health outcomes increased with the number of ACEs reported even after controlling for confounding variables. Thus, those who experienced 4+ ACEs were found to have between a three- and seven-fold increase in odds of reporting these sexual health outcomes compared to those with no ACEs. These findings align with international research, suggesting that ACEs increase the risk of certain sexual health outcomes, including the early initiation of sex, teenage pregnancy, multiple sexual partners [21], early puberty [22], unprotected sex [23,24], and repeat abortions [25]. The findings from this large UK sample contribute to the growing international evidence base on the relationship between ACEs and sexual health outcomes, which is overwhelmingly dominated by studies conducted in the US.

There may be a number of mechanisms by which ACEs are linked to participating in risky sexual behaviours. Toxic stress from ACE exposure in the absence of a caring adult is known to impact on a child’s neurological and biological functioning [29]. Whilst these changes are thought to be adaptive strategies, enabling the growing child to survive and reproduce in adverse circumstances [30], they can have lifelong impacts on perceptions, thoughts, and behaviours. Links between ACEs and risky sexual behaviour can be mediated by emotional dysregulation (difficulties regulating one’s emotions), whereby risky sexual behaviours (and other health-risk behaviours) are used as a way of coping with stressful experiences [31,32]. Exposure to ACEs may also be indicative of less parental control, supervision, or knowledge of children’s whereabouts in daily life, which can increase the likelihood of risky sexual behaviours in adolescence [33]. Risky sexual behaviours may also emerge as children who lack supportive and loving relationships at home seek to achieve these elsewhere [34]. ACEs can also impact on self-esteem and self-respect, leaving children vulnerable to sexual exploitation. For some young women, motherhood may present an opportunity to develop a socially valued identity and sense of self-worth [35]. ACEs may also increase the likelihood of other health risk behaviours, such as alcohol or drug use [36] that in turn may increase the likelihood of risky sexual behaviour.

The findings here indicate that being born to a teenage mother increased the risk of a number of poor sexual health outcomes for both genders, suggesting an intergenerational component to these outcomes. Our findings correspond with international studies, which show that being born to a teenage mother increases the likelihood of risky sexual behaviours, including early sexual initiation and adolescent births [37]. Furthermore, having a teenage mother has been associated with a plethora of poorer health outcomes for the child in the short term, including preterm delivery, low birth weight, small for gestational age, and neonatal and infant mortality, as well as increased risk of developmental and behavioural problems [38,39], which could impact on sexual behaviours. However, links between having a teenage mother and risky sexual behaviour may also, in part, be biological (e.g., similar age of menarche) and social (e.g., shared attitudes and beliefs around parenthood) [27]. Our findings place emphasis on the need to support young people to make informed choices around teenage parenthood, and to support young parents through maternity, early years, and beyond to improve parenting skills and health outcomes of both young parents and their offspring.

The odds of having early sex, becoming accidentally pregnant (for females only), and having a lifetime STI diagnosis were higher across younger age groups. Although of interest, these findings are likely a reflection of changes in societal attitudes towards sex and sexual behaviour [40]. Findings here indicate that, compared to those of white ethnicity, individuals of Asian ethnicity were significantly less likely to experience all four sexual health outcomes, except for males where being a teenage parent failed to reach significance. These findings align with previous data from the UK that has shown that individuals from Indian and Pakistani ethnic groups exhibit less sexual risk-taking behaviour than other ethnic groups [41].

International research has highlighted strong links between higher levels of deprivation and higher rates of teenage pregnancy [42,43] and increased STI diagnoses [44]. Here, being a teenage parent, having early sex (males only), and having an accidental teenage pregnancy (females only) were associated with increased deprivation in adulthood, although participants’ childhood socioeconomic status was not measured. Although links have been found between childhood and adulthood poverty, suggesting that deprivation can persist across generations [45], an additional measure of childhood socio-economic status may help to better understand the links between deprivation and sexual health outcomes. Our findings suggested that for women currently living in the most deprived areas (compared to those in the least deprived areas), there was a two-fold increase in the risk of having had an accidental pregnancy before the age of 18, whilst the risk of having become a teenage parent was six-fold. These findings could reflect different cultural beliefs and attitudes towards sex and pregnancy held within different socio-demographic groups, or may reflect how teenage parenthood can impede future educational and employment opportunities. Findings may also be indicative of limited relationship and sex education, sexual health services or support in more deprived areas, or a difference in access and uptake of such services. ACE exposure is often highest for those growing up in deprived areas [46]. However, even when including deprivation in our model as a confounder, negative sexual health outcomes were independently associated with ACE exposure. These findings may have significance for educational and other services related to sexual health which operate in more deprived areas, who may see a higher proportion of individuals who have been exposed to ACEs. Such services may provide a source of resilience to individuals in coping with challenging circumstances.

Study location was significantly associated with early sex, having a teenage pregnancy, and becoming a teenage parent, for both genders. Whilst we are unable to determine why these outcomes varied across location, rates of teenage pregnancy have been found to be related to social factors such as levels of youth unemployment, community population composition, and housing affordability [47], and these social factors can vary across geographical regions of the UK.

The strong association between ACEs and poor sexual health outcomes identified here provides evidence for the need for effective interventions to both prevent and ameliorate the effects of ACEs across societies. This is particularly so given the high prevalence of ACEs reported across general populations in this and other international studies [48,49]. Across our four surveys, for instance, almost half of participants had experienced one or more ACEs and almost one in ten had experienced 4+ ACEs. Preventing and addressing ACEs in families could help to protect against poor sexual health and other health outcomes. This could include, e.g., nurse-led home-visiting programmes [50], which can provide support to new and vulnerable mothers, or parenting programmes [51], which aim to strengthen parenting skills and family communication. Supporting new parents, or those with young children, can be a useful entry point for intervention, potentially disrupting cycles of ACEs that can occur across generations.

There are a number of known protective factors for poor sexual health outcomes across individuals, families, and communities. At the individual level, strategies that are effective at teaching self-regulation may be beneficial [52]. Education also has a key role to play. It is important that schools provide an environment which is trauma-informed but also trauma-responsive, fostering adult–child relationships. A review of school-based educational programmes that aimed to prevent child sexual abuse found evidence of improvements in protective behaviours and knowledge of sexual abuse prevention among children, with increased odds for disclosure [53]. With risky sexual behaviours often beginning in adolescence, programmes that focus on strengthening childhood resilience to cope with adversity may also offer protection against the sexual health outcomes associated with ACEs examined here. There are a number of ways to build childhood resilience, including developing life skills and building healthy relationships. Having a trusted adult offers protection against a range of negative outcomes associated with ACEs [54]. Where children do not have a trusted adult at home, ensuring opportunities to develop trusted adult relationships in other settings is critical (e.g., school, clubs), and can help avoid any risk of exploitation if close relationships are sought by the child elsewhere. However, the impact of resilience on lessening the associations between ACEs and negative sexual health outcomes is underexplored. As such, this is an area that future research should investigate.

The provision of trauma-informed sexual health services (e.g., contraception, advice, and treatment), as well as antenatal and postnatal services (e.g., health visiting services) [55], particularly for teenagers and young parents, is essential given that risky sexual behaviours can be embedded in childhood trauma [29]. Trauma-informed services can provide safe spaces and relationships to begin conversations about ACEs and their effects on health. Through further support and referral, they can play an important role in helping individuals and families to address adversities, find healthy ways of coping, and ameliorate any longer-term impacts of ACEs for both individuals and future generations. In beginning to address the underlying issues associated with risky sexual behaviours, trauma-informed services could also help to reduce repeated sexual health service use. With both ACE exposure and poor sexual health outcomes higher in more deprived areas, a focus should be on ensuring that those resident in these areas have access to high-quality trauma-informed sexual health and antenatal/postnatal services, as well as addressing barriers to uptake in these areas. This is essential since individuals who have suffered ACEs may be less trusting of support services [56,57].

Although we investigated ACEs and sexual health outcomes in one of the largest samples of its kind in the UK, there a number of limitations which should be acknowledged. Sexual orientation was not collected in all surveys and therefore was not included in analyses, meaning that nuance around sexual orientation was likely lost. Two of the sexual health outcomes used related directly to pregnancy and heterosexual sex. Sexual behaviour differs among sexual orientation groups, with men who have sex with men at increased risk of HIV and STIs [58,59]. Furthermore, lesbian, gay, bisexual, and transgender (LGBT) populations are more likely to have experienced ACEs than heterosexual populations [60,61]. Levels of lifetime STI diagnoses may be under-reported amongst certain population groups, such as those living in more deprived areas or those experiencing ACEs, since diagnosis depends on having access to, or wishing to seek access to, STI testing and treatment. Deprivation was measured using IMD quintile and was based on current postcode, and we did not measure childhood deprivation. Although this may have confounded the links found between socio-economic factors and ACE exposure, it was considered the best available measure of participants’ deprivation. As with all cross-sectional surveys, our data provide a static snapshot of people’s lives, and measures of ACEs and other variables were based on participants’ recollection of past events. Whilst our cross-sectional surveys were useful for establishing potential links, prospective and longitudinal approaches may help to elucidate these relationships further. Data were combined across four studies to generate a large dataset for analysis, with data collected over different time points. This may have introduced differences in the way data were collected. However, study methodologies were similar and study location was controlled for in logistic regression analyses to address this. Finally, data were collected between 2013 and 2015. Socio-cultural changes relating to sexual behaviours and social norms, as well as changes in the availability of health services, may have occurred since data collection that may impact on the relationships between ACEs, demographics, and sexual health outcomes.

## 5. Conclusions

Exposure to ACEs is associated with a three- to seven-fold increase in the likelihood of having poor sexual health outcomes in adolescence and adulthood. Our findings highlight the need for effective interventions to both prevent ACEs and ameliorate their lifelong effects. Sexual health, antenatal/postnatal, and sex education services may benefit from taking a trauma-informed approach, helping individuals to understand and cope with the effects of current or past adverse experiences. In particular, services for teenagers and young parents may offer valuable windows of opportunity for both ACE prevention and support for those already affected. Work should continue to explore barriers to uptake of these services.

## Figures and Tables

**Table 1:** Prevalence of sexual health outcomes by participant demographics, ACE count, being born to a teenage mother, and study location.

		Total %	Early Sex < 16%	Accidental Teenage Pregnancy %	Teenage Parent %	Lifetime STI Diagnosis %
		*n*= 12,788	*n* = 11,412	*n* = 12,682	*n* = 12,524	*n* = 12,761
Gender	Male	45.1	19.0	4.7	2.0	2.0
	Female	54.9	14.5	9.0	8.5	1.6
	X^2^		41.835	86.849	251.448	3.171
	*p*		**<0.001**	**<0.001**	**<0.001**	0.075
Age group	18–29	22.7	25.7	8.8	6.4	2.5
	30–39	20.3	18.0	6.5	5.5	2.1
	40–49	20.3	15.5	7.6	5.2	1.5
	50–59	17.1	13.9	7.2	5.6	1.8
	60–69	19.6	6.8	4.9	5.2	0.7
	X^2^		330.069	33.479	5.205	27.807
	*p*		**<0.001**	**<0.001**	0.267	**<0.001**
Ethnicity	White	83.6	18.4	7.8	5.7	1.9
	Asian	11.3	3.0	1.0	5.0	0.3
	Other	5.1	13.7	7.6	4.8	2.0
	X^2^		184.080	90.856	1.955	18.949
	*p*		**<0.001**	**<0.001**	0.376	**<0.001**
Deprivation quintile	1 (least)	22.3	12.7	4.8	1.9	1.5
	2	18.7	15.1	7.1	3.4	1.9
	3	18.9	17.2	7.7	5.7	1.5
	4	19.2	16.1	7.0	6.3	1.8
	5 (most)	20.9	21.7	8.9	10.7	2.2
	X^2^		78.314	37.211	225.052	5.216
	*p*		**<0.001**	**<0.001**	**<0.001**	0.266
ACE count	0	55.9	10.3	3.9	3.9	0.8
	1	19.9	17.3	7.6	5.9	1.5
	2–3	15.0	23.2	9.3	6.6	2.9
	4+	9.2	38.6	21.1	13.4	6.7
	X^2^		629.145	474.789	175.556	221.674
	*p*		**<0.001**	**<0.001**	**<0.001**	**<0.001**
Born to teenage mother *	No	92.8	16.2	7.0	4.8	1.9
	Yes	7.2	26.4	13.7	15.9	2.9
	X^2^		49.783	45.745	162.402	3.465
	*p*		**<0.001**	**<0.001**	**<0.001**	0.063
Study location	England	30.4	16.8	5.5	5.6	2.5
	Wales	15.9	24.0	11.3	5.9	1.0
	S Eng	42.6	12.1	6.1	4.2	1.4
	NW Eng	11.1	20.8	8.7	10.5	2.2
	X^2^		165.990	83.693	84.344	22.414
	*p*		**<0.001**	**<0.001**	**<0.001**	**<0.001**

* *n* = 10,655, missing cases = 2133. STI = sexually transmitted infection; S Eng = South England; NW Eng = North West England. Values in bold indicate statistical significance.

**Table 2:** Adjusted odds ratios for sexual health outcomes: males.

		Early Sex < 16				Accidental Teenage Pregnancy				Teenage Parent				Lifetime STI Diagnosis			
		AOR	LCI	UCI	*p*	AOR	LCI	UCI	*p*	AOR	LCI	UCI	*p*	AOR	LCI	UCI	*p*
ACE count	0	Ref			**<0.001**	Ref			**<0.001**	Ref			**0.002**	Ref			**<0.001**
	1	1.427	1.157	1.759	0.001	1.712	1.165	2.517	0.006	1.742	0.989	3.066	0.055	1.447	0.792	2.641	0.229
	2–3	2.283	1.845	2.825	<0.001	2.254	1.529	3.321	<0.001	1.994	1.122	3.543	0.019	2.492	1.416	4.385	0.002
	4+	3.224	2.499	4.158	<0.001	6.167	4.254	8.942	<0.001	3.161	1.730	5.776	<0.001	7.646	4.425	13.211	<0.001
Age group (years)	18–29	3.401	2.575	4.491	<0.001	1.177	0.740	1.871	0.491	0.436	0.233	0.817	0.010	2.315	1.114	4.811	0.024
	30–39	2.548	1.885	3.445	<0.001	1.619	1.002	2.618	0.049	0.648	0.339	1.235	0.187	2.447	1.154	5.188	0.020
	40–49	2.111	1.564	2.850	<0.001	1.509	0.937	2.431	0.091	0.590	0.310	1.122	0.108	1.619	0.733	3.571	0.233
	50–59	1.974	1.453	2.683	<0.001	1.391	0.851	2.274	0.188	0.605	0.312	1.172	0.136	1.509	0.667	3.413	0.323
	60–69	Ref			**<0.001**	Ref			0.245	Ref			0.132	Ref			0.103
Ethnicity	White	Ref			**<0.001**	Ref			**0.003**	Ref			0.459	Ref			0.075
	Asian	0.147	0.092	0.235	<0.001	0.208	0.084	0.517	0.001	0.611	0.254	1.467	0.270	0.259	0.081	0.835	0.024
	Other	0.697	0.468	1.039	0.076	0.953	0.509	1.784	0.881	1.240	0.523	2.939	0.625	0.851	0.356	2.037	0.718
Deprivation quintile	1 (least)	Ref			**<0.001**	Ref			0.128	Ref			**0.003**	Ref			0.551
	2	1.356	1.043	1.762	0.023	1.636	1.042	2.570	0.032	1.847	0.749	4.560	0.183	1.298	0.697	2.418	0.410
	3	1.617	1.248	2.095	<0.001	1.761	1.124	2.759	0.014	3.599	1.583	8.180	0.002	0.805	0.399	1.623	0.544
	4	1.466	1.122	1.917	0.005	1.422	0.887	2.280	0.144	3.483	1.512	8.026	0.003	1.374	0.733	2.575	0.322
	5 (most)	1.993	1.531	2.594	<0.001	1.578	0.990	2.517	0.055	4.452	1.955	10.137	<0.001	1.107	0.564	2.172	0.768
Born to teen mum	No	Ref				Ref				Ref				Ref			
	Yes	1.597	1.187	2.148	**0.002**	1.332	0.838	2.117	0.225	2.768	1.584	4.837	**<0.001**	1.032	0.498	2.139	0.933
Study location	England	Ref			**<0.001**	Ref			**<0.001**	Ref			**0.021**	Ref			**<0.001**
	Wales	1.362	1.090	1.701	0.007	2.838	1.938	4.155	<0.001	2.512	1.381	4.567	0.003	0.154	0.065	0.365	<0.001
	S Eng	0.684	0.559	0.836	<0.001	1.276	0.876	1.858	0.204	1.545	0.875	2.727	0.134	0.483	0.308	0.760	0.002
	NW Eng	1.344	1.007	1.795	0.045	1.663	0.981	2.819	0.059	1.269	0.576	2.799	0.554	0.483	0.223	1.048	0.065

AOR = adjusted odds ratio; LCI = lower confidence interval; UCI = upper confidence interval; Ref = reference category; STI = sexually transmitted infection; S Eng = South England; NW Eng = North West England. Values in bold indicate statistical significance.

**Table 3:** Adjusted odds ratios for sexual health outcomes: females.

		Early Sex <16				Accidental Teenage Pregnancy				Teenage Parent				Lifetime STI Diagnosis			
		AOR	LCI	UCI	*p*	AOR	LCI	UCI	*p*	AOR	LCI	UCI	*p*	AOR	LCI	UCI	*p*
ACE count	0	Ref			**<0.001**	Ref			**<0.001**	Ref			**<0.001**	Ref			**<0.001**
	1	1.514	1.226	1.871	<0.001	1.753	1.377	2.232	<0.001	1.613	1.254	2.075	<0.001	1.692	0.916	3.125	0.093
	2-3	2.187	1.760	2.716	<0.001	2.008	1.557	2.589	<0.001	1.626	1.235	2.141	0.001	3.324	1.899	5.815	<0.001
	4+	4.271	3.425	5.327	<0.001	4.296	3.358	5.495	<0.001	3.190	2.439	4.172	<0.001	7.138	4.232	12.040	<0.001
Age group (years)	18–29	6.339	4.529	8.874	<0.001	1.709	1.261	2.316	0.001	1.191	0.872	1.627	0.272	3.428	1.412	8.323	0.006
	30–39	4.514	3.198	6.373	<0.001	1.169	0.846	1.616	0.344	1.036	0.749	1.432	0.831	3.143	1.285	7.687	0.012
	40–49	3.142	2.212	4.465	<0.001	1.400	1.023	1.917	0.035	0.952	0.684	1.326	0.773	1.884	0.738	4.809	0.185
	50–59	2.477	1.706	3.596	<0.001	1.390	0.999	1.935	0.051	1.212	0.863	1.702	0.268	3.417	1.376	8.482	0.008
	60–69	Ref			**<0.001**	Ref			**0.005**	Ref			0.426	Ref			**0.022**
Ethnicity	White	Ref			**<0.001**	Ref			**<0.001**	Ref			**0.005**	Ref			0.077
	Asian	0.057	0.027	0.121	<0.001	0.076	0.031	0.185	<0.001	0.611	0.426	0.877	0.008	0.197	0.047	0.816	0.025
	Other	0.467	0.295	0.739	0.001	1.008	0.662	1.536	0.970	0.588	0.351	0.984	0.043	1.112	0.470	2.628	0.809
Deprivation quintile	1 (least)	Ref			0.138	Ref			**0.001**	Ref			**<0.001**	Ref			0.979
	2	1.012	0.784	1.307	0.925	1.397	1.032	1.892	0.031	2.160	1.403	3.327	<0.001	1.005	0.544	1.857	0.988
	3	1.165	0.901	1.505	0.244	1.743	1.292	2.352	<0.001	3.812	2.536	5.733	<0.001	0.878	0.454	1.699	0.699
	4	1.091	0.845	1.409	0.505	1.531	1.130	2.075	0.006	3.334	2.209	5.031	<0.001	0.874	0.463	1.648	0.677
	5 (most)	1.346	1.043	1.738	0.023	1.839	1.353	2.500	<0.001	6.395	4.294	9.524	<0.001	1.019	0.546	1.903	0.953
Born to teen mum	No	Ref				Ref				Ref				Ref			
	Yes	1.717	1.322	2.230	**<0.001**	1.855	1.403	2.452	**<0.001**	2.931	2.262	3.797	**<0.001**	1.308	0.708	2.415	0.391
Study location	England	Ref			**<0.001**	Ref			**<0.001**	Ref			**0.003**	Ref			0.120
	Wales	1.622	1.289	2.040	<0.001	1.950	1.484	2.562	<0.001	0.901	0.663	1.224	0.505	0.581	0.292	1.156	0.122
	S Eng	0.839	0.688	1.024	0.085	1.411	1.120	1.779	0.004	0.865	0.678	1.103	0.241	0.752	0.473	1.196	0.229
	NW Eng	1.517	1.168	1.970	0.002	1.762	1.296	2.397	<0.001	1.468	1.107	1.947	0.008	1.347	0.744	2.441	0.325

AOR = adjusted odds ratio; LCI = lower confidence interval; UCI = upper confidence interval; Ref = reference category; STI = sexually transmitted infection; S Eng = South England; NW Eng = North West England. Values in bold indicate statistical significance.

## Data Availability

The data are not publicly available due to privacy and ethical restrictions.

## Supplementary Materials

The following supporting information can be downloaded at: https://www.mdpi.com/article/10.3390/ijerph19148869/s1, Table S1: Individual survey sample characteristics; Table S2: Adverse childhood experiences, born to a teenage mother and sexual health outcomes. References [20,62,63,64] are cited in the supplementary materials.
