# Supplementary material for: Adverse Childhood Experiences and Their Relationship with Poor Sexual Health Outcomes: Results from Four Cross-Sectional Surveys
Source: Int J Environ Res Public Health. 2022 Jul 21;19(14):8869. doi: 10.3390/ijerph19148869 (PMC9316235; doi:10.3390/ijerph19148869)
Supplement: Supplementary file 1 [file ijerph-19-08869-s001.zip › ijerph-1806053-supplementary.pdf]

**Table S1.** Individual survey sample characteristics.

| Country | Study location | Stratification for sampling | Dates | Age Range (years) | Final sample[d] (n) | Compliance[e] | Deprivation Measure | Reference |
|---|---|---|---|---|---|---|---|---|
| England | North West England | Blackburn with Darwen Local Authority[a] | 2012 (Aug-Sep) | 18-70[c] | 1500 | 70.4% | IMD 2010 | [1] |
| England | National sample | English Administrative Regions (n=10)[b] | 2013 (Apr-Jul) | 18-69 | 4010 | 53.5% | IMD 2010 | [2] |
| England | South of England | Luton, Hertfordshire Northamptonshire (n=3) | 2015 (Jun-Sep) | 18-69 | 5623 | 55.8% | IMD 2011 | [3] |
| Wales | National sample | Welsh Health Regions (n=7) | 2015 (Feb-May) | 18-69 | 2028 | 49.1% | WIMD 2014 | [4] |

[a]No sub-regional stratification was undertaken in Blackburn with Darwen due to the relatively small size of the sample area. [b]London was split into Inner and Outer London for regional sampling. [c]Individuals aged 70 years were excluded from the final sample (see methods). IMD = Index of Multiple Deprivation; WIMD = Welsh Index of Multiple Deprivation. [d]Sample sizes were determined for each study area in order to ensure, for analytical purposes, adequate representation of individuals with higher ACE counts. [e]Individuals not completing all questions on variables of interest were excluded.

References

(1) Bellis, M.A., Lowey, H., Leckenby, N., Hughes, K., Harrison, D. Adverse childhood experiences: retrospective study to determine their impact on adult health behaviours and health outcomes in a UK population, J Public Health, 2014, 36 (1), 81–91.
(2) Bellis, M. A.; Hughes, K.; Leckenby, N.; Hardcastle, K. A.; Perkins, C.; Lowey, H. Measuring mortality and the burden of adult disease associated with adverse childhood experiences in England: a national survey. J Public Health, 2015, 37 (3), 445–454.
(3) Ford K, Butler N, Hughes K, Bellis MA. Adverse childhood experiences (ACEs) in Herfordshire, Luton and Northamptonshire. Liverpool: Liverpool John Moores University, 2016.
(4) Bellis, M.A., Ashton, K., Hughes, K., Ford, K., Bishop, J., Paranjothy, S. Adverse childhood experiences and their impact on health-harming behaviours in the Welsh adult population. Cardiff: Public Health Wales, 2015.

**Table S2.** Adverse childhood experiences, born to a teenage mother and sexual health outcomes.

| **ACE questions.** All ACE questions were preceded by the statement "While you were growing up, before the age of 18..." | | |
|---|---|---|
| **ACE** | **Question** | **Qualifying response** |
| *Physical abuse* | How often did a parent or adult in your home ever hit, beat, kick, or physically hurt you in any way? This does not include gentle smacking for punishment? | Once or more than once |
| *Verbal abuse* | How often did a parent or adult in your home ever swear at you, insult you, or put you down? | More than once |
| *Sexual abuse* | How often did anyone at least 5 years older than you (including adults) ever touch you sexually? | Once or more than once to any of the questions |
| | How often did anyone at least 5 years older than you (including adults) try to make you touch them sexually? | |
| | How often did anyone at least 5 years older than you (including adults) force you to have any type of sexual intercourse (oral, anal, or vaginal)? | |
| *Parental separation* | Were your parents ever separated or divorced? | Yes |
| *Domestic violence* | How often did your parents or adults in your home ever slap, hit, kick, punch, or beat each other up? | Once or more than once |
| *Mental illness* | Did you live with anyone who was depressed, mentally ill, or suicidal? | Yes |
| *Alcohol abuse* | Did you live with anyone who was a problem drinker or alcoholic? | Yes |
| *Drug abuse* | Did you live with anyone who used illegal street drugs or who abused prescription medications? | Yes |
| *Incarceration* | Did you live with anyone who served time or was sentenced to serve time in a prison or young offenders' institution? | Yes |
| **Teenage mother** | | |
| *Born to teenage mother* | Approximately how old was your mother when you were born? (age years) | Aged 18 or less |
| **Sexual health outcomes** | | |
